# 2192: Comparison of liquid Versus dry aerosol drug delivery in a 3D printed avian trachea and mainstream bronchi model

**DOI:** 10.1017/cts.2017.27

**Published:** 2018-05-10

**Authors:** Carlos Abraham Ruvalcaba, Roger Monroy, Lisa A. Tell, Christine V. Fiorello, Jerold Last, Jean-Pierre Delplanque

**Affiliations:** University of California, Davis, CA, USA

## Abstract

OBJECTIVES/SPECIFIC AIMS: This study investigates the process configuration parameters involved in targeted drug delivery to the avian respiratory system. Previously, direct intratracheal aerosol delivery in an avian model using a commercial atomizer was found to result in delivery of a high portion of the total dose into one lung lobe. We hypothesize that controlling process configuration will decrease the asymmetric distribution. METHODS/STUDY POPULATION: A 3D printed model of an avian trachea and mainstream bronchi was constructed to create a representative model for direct instillation of aerosols. Construction of the model respiratory tract included the trachea and the first mainstream bronchi bifurcation to measure left/right (L/R) distribution of aerosol delivered. Both liquid aerosol delivery (LAD) using a commercial atomizer and dry aerosol delivery (DAD) using a custom-built dry powder insufflator device were tested. Two experimental variables were controlled: (1) retraction distance from the carina and (2) centering of device shaft in the lumen of the trachea. Measurement of device efficiency (dose delivered to the 3D model at as fraction of total dose), aerosol delivery efficiency (dose captured at L/R bifurcations as a fraction of total dose), and aerosol lateralization (L/R) was conducted. RESULTS/ANTICIPATED RESULTS: The aerosol delivery efficiency for both LAD and DAD devices [73.9% (95% CI: 68.2–79.2) and 73.4% (95% CI: 55.5–91.3), respectively] did not have an appreciable difference. However, the LAD device had a higher efficiency as compared with the DAD device. The L/R distribution for the DAD device was found to be highly dependent on both retraction distance and shaft centering. Appreciable improvement in the L/R distribution was seen using the DAD device by increasing the retraction distance distal to the carina. DISCUSSION/SIGNIFICANCE OF IMPACT: The use of targeted drug delivery to treat pulmonary pathogens requires a careful design, manufacture, and therapeutic positioning of devices. In particular, clinically relevant animal models and treatment regimes requires a sound understanding of the physical processes controlling aerosol distribution in the respiratory system. By using a simulated respiratory model, many of the physical parameters of drug delivery can be tested before using a live animal model. This is especially important from an animal welfare perspective as well as an animal subject availability aspect.

